# Postoperative superior mesenteric artery stenting following pancreaticoduodenectomy in a patient with severe mesenteric atherosclerosis

**DOI:** 10.1093/jscr/rjag124

**Published:** 2026-03-29

**Authors:** Ilias P Doulamis, Victor Molina, Jerry Zhang, Coleen Rizzo, Aarathi Minisandram, Attila Nakeeb, Michael Levy, Jeffrey Indes

**Affiliations:** Division of Vascular Surgery, Lahey Clinic, 41 Mall Rd, Burlington, MA 01805, United States; Department of Surgery, Lahey Clinic, 41 Mall Rd, Burlington, MA 01805, United States; Department of Cardiology, Lahey Clinic, 41 Mall Rd, Burlington, MA 01805, United States; Division of Vascular Surgery, Lahey Clinic, 41 Mall Rd, Burlington, MA 01805, United States; Department of Surgery, Lahey Clinic, 41 Mall Rd, Burlington, MA 01805, United States; Division of Vascular Surgery, Lahey Clinic, 41 Mall Rd, Burlington, MA 01805, United States; Department of Surgery, Lahey Clinic, 41 Mall Rd, Burlington, MA 01805, United States; Division of Vascular Surgery, Lahey Clinic, 41 Mall Rd, Burlington, MA 01805, United States; Department of Surgery, Lahey Clinic, 41 Mall Rd, Burlington, MA 01805, United States; UMass Chan Medical School, 55 N Lake Ave, Worcester, MA 01655, United States; Department of Cardiology, Lahey Clinic, 41 Mall Rd, Burlington, MA 01805, United States; UMass Chan Medical School, 55 N Lake Ave, Worcester, MA 01655, United States; Division of Vascular Surgery, Lahey Clinic, 41 Mall Rd, Burlington, MA 01805, United States; UMass Chan Medical School, 55 N Lake Ave, Worcester, MA 01655, United States

**Keywords:** superior mesenteric artery occlusion, stenting, pancreaticoduodenectomy, acute mesenteric ischemia

## Abstract

Pancreaticoduodenectomy (PD) in patients with severe mesenteric atherosclerosis carries a risk of hepatic and intestinal ischemia after ligation of the gastroduodenal artery (GDA). We report the case of a 69-year-old male with pancreatic head adenocarcinoma who underwent PD. Intraoperatively, hepatic artery flow was only pressure-dependent after GDA division. Postoperatively, he developed abdominal pain and emesis. Computerized tomography angiogram showed near-occlusive celiac and thrombosed superior mesenteric artery (SMA) with inferior mesenteric artery (IMA) collateralization. SMA stenting via the left brachial artery restored flow and resolved symptoms. This case underscores the importance of assessing mesenteric perfusion and considering early SMA stenting in high-risk patients prior to PD.

## Introduction

Pancreaticoduodenectomy (PD) remains the definitive treatment for periampullary and distal bile duct malignancies. In patients with diffuse atherosclerotic disease, hepatic and mesenteric perfusion may depend heavily on collateral pathways through the gastroduodenal artery (GDA). Ligation of the GDA, an essential step of PD, can therefore unmask hemodynamically significant stenosis of the celiac trunk or superior mesenteric artery (SMA), leading to hepatic or intestinal ischemia [1].

Preoperative vascular imaging and early recognition of compromised mesenteric flow are essential. Endovascular stenting of the SMA has emerged as a minimally invasive option to restore perfusion when ischemia is detected either preoperatively, intraoperatively, or postoperatively [2]. Herein, we report a case of delayed SMA stenting in a patient who developed postoperative SMA thrombosis following PD in a patient with extensive preoperative mesenteric atherosclerosis, resulting in reversal of ischemic symptoms and successful recovery.

## Case report

A 69-year-old male with type 2 diabetes, chronic obstructive pulmonary disease (COPD), hypertension, hyperlipidemia, a 90-pack-year smoking history, and daily alcohol use presented with progressive jaundice, vague abdominal pain, and a 50-lb weight loss over 5 months. Endoscopic retrograde cholangiopancreatography identified a tight distal common bile duct (CBD) stricture that was stented. Magnetic resonance imaging (MRI) showed an 8-mm high-grade distal CBD stricture, a 1.2-cm pancreatic head cystic lesion, and pancreatic body/tail atrophy. Due to persistent obstructive symptoms and concern for malignancy, he was scheduled for PD. Preoperative computerized tomography (CT) revealed heavily calcified celiac and SMA vessels, though inflow was not further assessed with CTA or duplex.

At exploration, hepatic artery pulsatility significantly decreased after GDA division when systolic pressure fell below 120 mmHg. Intraoperative Doppler confirmed diminished but present flow, and no vascular intervention was performed. Resection proceeded with pancreaticojejunostomy, hepaticojejunostomy, and gastrojejunostomy.

**Figure 1:**
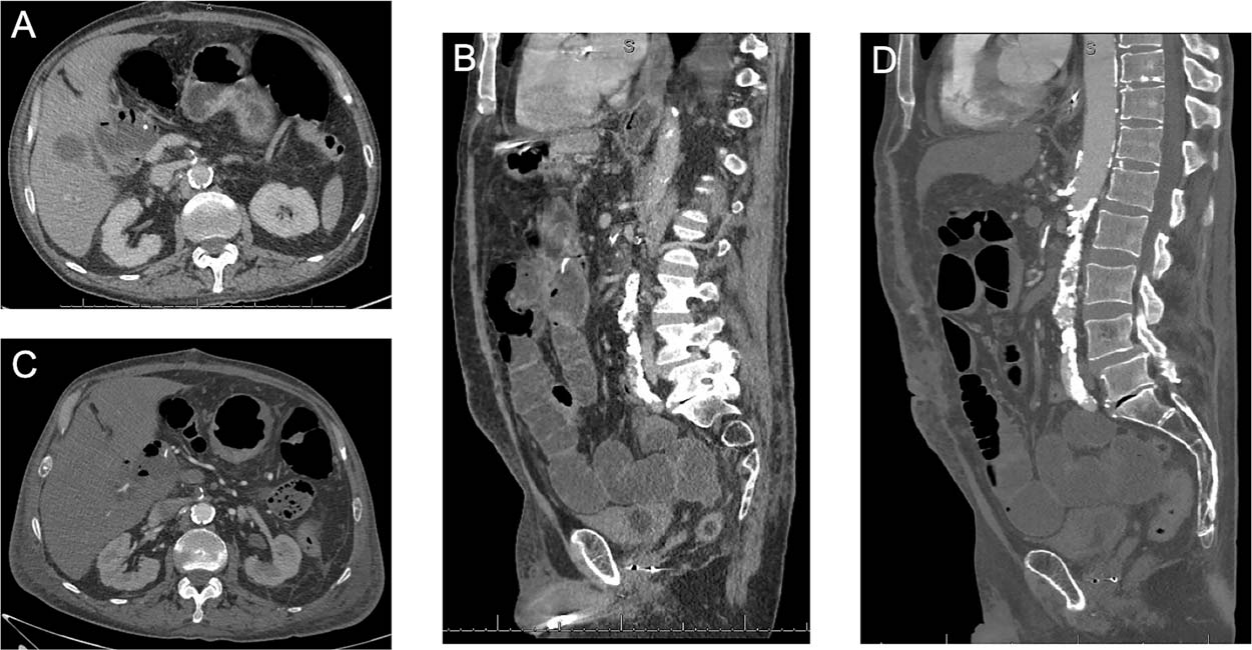
**(**A–B) Axial and coronal CT images showing the diseased celiac and SMA with flow at POD #10. (C–D) Axial and coronal CT images showing the diseased SMA with a new occlusive thrombus at POD #12.

The patient was extubated on postoperative day (POD) 2 and resuscitated for persistent lactic acidosis. He transferred to step-down on POD 3, started TPN on POD 4, and advanced to a regular diet by POD 7. Over subsequent days, he developed worsening abdominal pain and emesis. CT with intravenous (IV) contrast on POD 10 showed no ischemia (Fig. 1A–B). CTA on POD 12 revealed near-occlusive celiac stenosis and new occlusive SMA thrombosis with presumed retrograde inferior mesenteric artery (IMA) collateralization, but no bowel ischemia (Fig. 1C–D).

**Figure 2:**
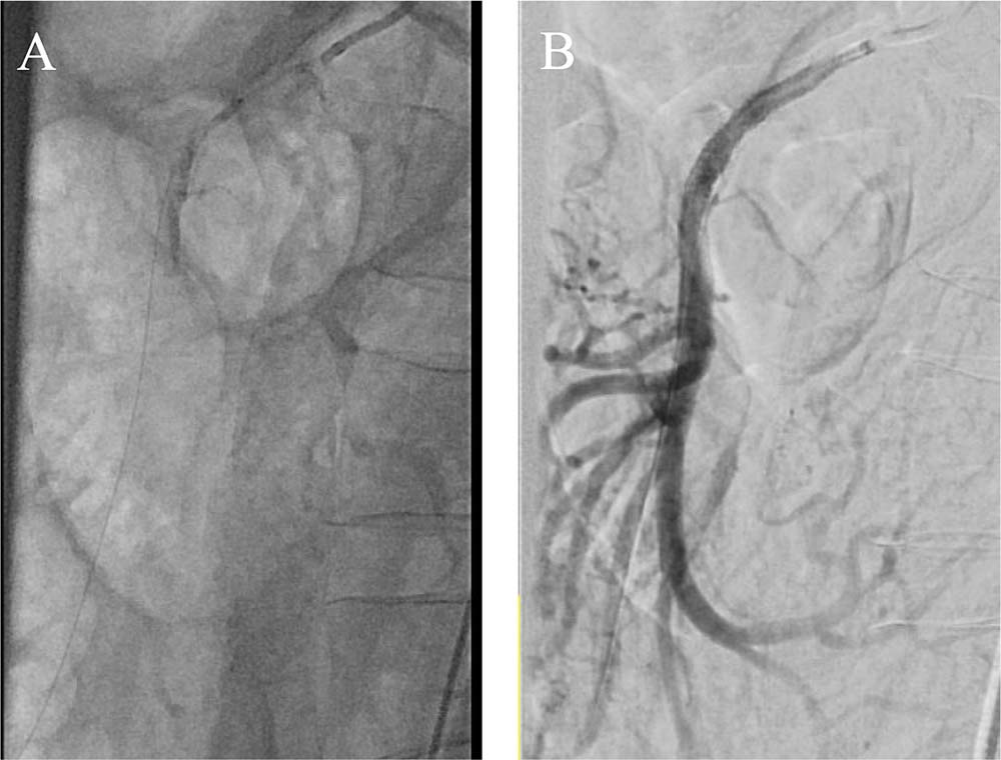
(A) Aortogram with wire passed through the SMA and (B) completion angiogram after stent placement.

Concern for evolving acute-on-chronic mesenteric ischemia prompted percutaneous SMA stenting on POD 14 via a left brachial approach, chosen due to calcified iliac arteries and weak femoral pulses. A 4 × 38 mm balloon-expandable drug-eluting stent (Resolute Onyx, Medtronic) was deployed across the SMA origin with restoration of antegrade flow and preserved branch perfusion (Fig. 2).

He was started on dual antiplatelet therapy and improved rapidly: lactic acidosis resolved within 24 hours, bowel function returned, and oral intake resumed. A brachial artery pseudoaneurysm developed but resolved after thrombin injection. TPN was discontinued by POD 20, and he was discharged to rehabilitation on POD 27.

## Discussion

This case highlights a critical but underrecognized cause of postoperative morbidity following PD, which is mesenteric hypoperfusion secondary to underestimated SMA and celiac atherosclerotic stenosis. Although there have been previous reports on celiac revascularization, given the concern of blood supply to the liver post-operatively, there has not been particular focus on SMA disease and intestinal ischemia since retrograde flow from the IMA is presumed to be sufficient [1].

**Figure 3:**
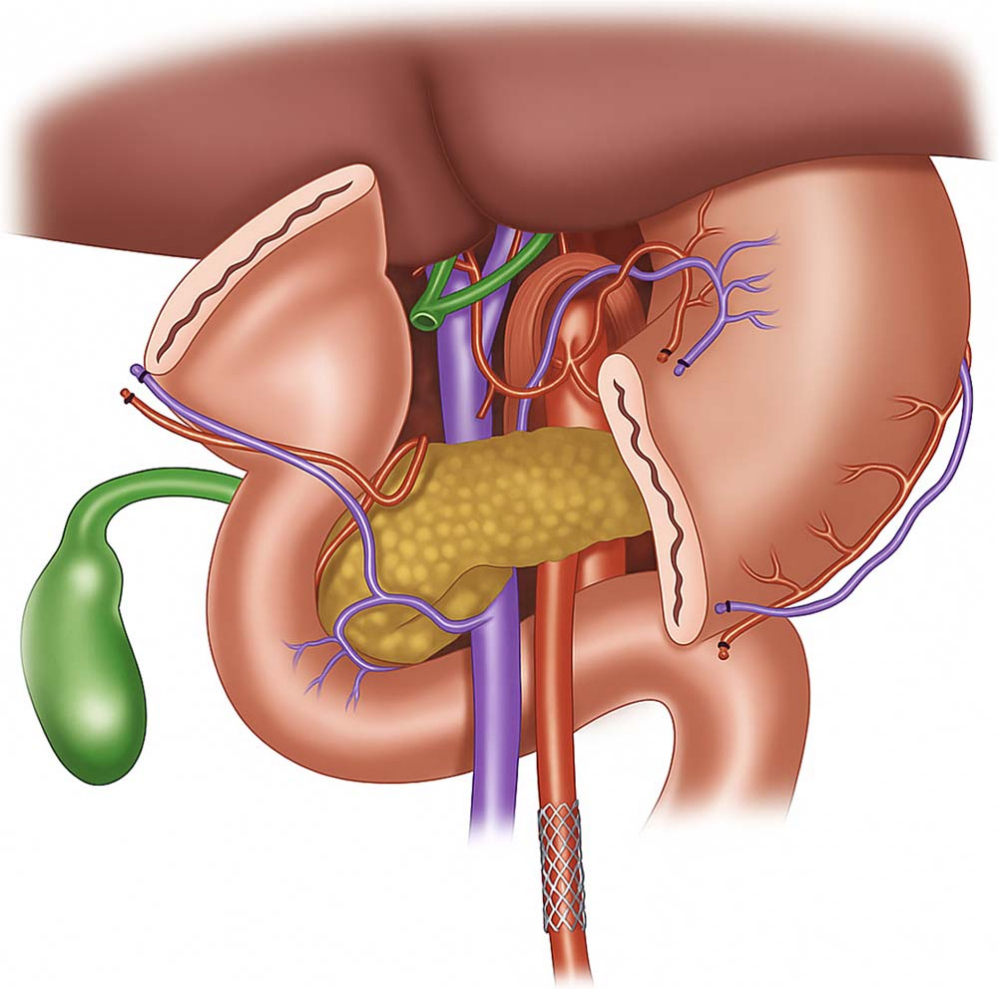
Illustration of stent in the SMA after division of the stomach for better visualization. This figure depicts the anatomy prior to Whipple resection. The stent is also more distal than our ostial lesion for better visualization purposes.

Preoperative vascular imaging and a focused history are essential in such patients. CTA or duplex can identify significant stenoses of the SMA or celiac trunk and help plan revascularization. When severe inflow compromise is detected, preoperative or staged endovascular stenting should be considered. SMA stenting before PD has been previously reported with good outcomes [3, 4] (Fig. 3). Antiplatelet therapy after stent placement must be weighed carefully, as it can increase bleeding risk during a complex operation like PD. Decisions about early revascularization should be individualized. If stenosis is identified intraoperatively, urgent vascular consultation and angiography can guide immediate management. When preoperative CTA is available, retrograde SMA stenting via distal exposure is an option. For celiac stenosis, median arcuate ligament release may improve flow, but it was not suitable in this case due to heavy ostial calcification.

Postoperative ischemia presents more insidiously. Lactic acidosis and post-prandial pain should raise suspicion for hypoperfusion. In our patient, CTA demonstrated thrombosis of the already heavily calcified SMA, explaining the symptoms of acute-on-chronic mesenteric ischemia. Endovascular SMA stenting restored antegrade mesenteric flow and resulted in rapid clinical recovery. In a similar scenario published by Tagkalos *et al.*, recanalization of the SMA was unsuccessful, but the patient was able to have a good recovery with anticoagulation only [1].

In more extensive disease, lithotripsy or simultaneous PD with antegrade or retrograde SMA bypass could be considered; however, the morbidity of such an operation could be significantly higher. Thus, it should be considered only in carefully selected individuals. A case of a patient with an occluded SMA undergoing PD not needing revascularization has been reported [5].

A bare-metal stent was used in this case to restore SMA flow. While bare-metal stents are easy to deploy and useful in urgent settings, growing evidence suggests covered stents offer better long-term patency, with lower restenosis rates and reduced recurrence of mesenteric ischemia. Although anatomy and device availability influence choice, covered stents are increasingly favored when durable SMA patency is the primary goal [6].

This case underscores the importance of early multidisciplinary collaboration between hepatopancreatobiliary and vascular surgery teams to prevent ischemic injury and reduce postoperative morbidity. While transfemoral access is standard, extensive iliofemoral disease or challenging SMA angulation may necessitate upper-extremity access for precise ostial stent placement. In this patient, radial access or a surgical cutdown might have avoided the postoperative brachial artery pseudoaneurysm. Balloon-expandable stents offer superior radial strength and deployment accuracy in heavily calcified proximal SMA lesions.

## Conclusion

Patients undergoing PD with known or suspected mesenteric disease require careful preoperative vascular assessment. In cases where ischemia develops postoperatively, endovascular SMA stenting provides safe and effective salvage therapy. Early intervention can restore perfusion, prevent intestinal necrosis, and significantly improve postoperative recovery.

## Declaration of generative AI and AI-assisted technologies in the manuscript preparation process

During the preparation of this work, the authors used ChatGPT 4.0 in order to create Fig. 3 and to proof check the manuscript. After using this tool/service, the authors reviewed and edited the content as needed and take full responsibility for the content of the published article.
